# Comparative Chloroplast Genome Analyses of Streptophyte Green Algae Uncover Major Structural Alterations in the Klebsormidiophyceae, Coleochaetophyceae and Zygnematophyceae

**DOI:** 10.3389/fpls.2016.00697

**Published:** 2016-05-24

**Authors:** Claude Lemieux, Christian Otis, Monique Turmel

**Affiliations:** Institut de Biologie Intégrative et des Systèmes, Département de Biochimie, de Microbiologie et de Bio-informatique, Université Laval, QuébecQC, Canada

**Keywords:** Streptophyta, charophytes, phylogenomics, plastid DNA, large inverted repeat, chloroplast genome evolution, genome rearrangements, group II introns

## Abstract

The Streptophyta comprises all land plants and six main lineages of freshwater green algae: Mesostigmatophyceae, Chlorokybophyceae, Klebsormidiophyceae, Charophyceae, Coleochaetophyceae and Zygnematophyceae. Previous comparisons of the chloroplast genome from nine streptophyte algae (including four zygnematophyceans) revealed that, although land plant chloroplast DNAs (cpDNAs) inherited most of their highly conserved structural features from green algal ancestors, considerable cpDNA changes took place during the evolution of the Zygnematophyceae, the sister group of land plants. To gain deeper insights into the evolutionary dynamics of the chloroplast genome in streptophyte algae, we sequenced the cpDNAs of nine additional taxa: two klebsormidiophyceans (*Entransia fimbriata* and *Klebsormidium* sp. SAG 51.86), one coleocheatophycean (*Coleochaete scutata*) and six zygnematophyceans (*Cylindrocystis brebissonii, Netrium digitus, Roya obtusa, Spirogyra maxima, Cosmarium botrytis* and *Closterium baillyanum*). Our comparative analyses of these genomes with their streptophyte algal counterparts indicate that the large inverted repeat (IR) encoding the rDNA operon experienced loss or expansion/contraction in all three sampled classes and that genes were extensively shuﬄed in both the Klebsormidiophyceae and Zygnematophyceae. The klebsormidiophycean genomes boast greatly expanded IRs, with the *Entransia* 60,590-bp IR being the largest known among green algae. The 206,025-bp *Entransia* cpDNA, which is one of the largest genome among streptophytes, encodes 118 standard genes, i.e., four additional genes compared to its *Klebsormidium flaccidum* homolog. We inferred that seven of the 21 group II introns usually found in land plants were already present in the common ancestor of the Klebsormidiophyceae and its sister lineages. At 107,236 bp and with 117 standard genes, the *Coleochaete* IR-less genome is both the smallest and most compact among the streptophyte algal cpDNAs analyzed thus far; it lacks eight genes relative to its *Chaetosphaeridium globosum* homolog, four of which represent unique events in the evolutionary scenario of gene losses we reconstructed for streptophyte algae. The 10 compared zygnematophycean cpDNAs display tremendous variations at all levels, except gene content. During zygnematophycean evolution, the IR disappeared a minimum of five times, the rDNA operon was broken at four distinct sites, group II introns were lost on at least 43 occasions, and putative foreign genes, mainly of phage/viral origin, were gained.

## Introduction

The green plants, also referred to as Viridiplantae or Chloroplastida, split early (1200-700 Mya) into two main clades, the Chlorophyta and Streptophyta (Lewis and McCourt, 2004; Leliaert et al., 2012; Becker, 2013), and about 470 Mya, green algae from the Streptophyta gave rise to all land plants (Gensel, 2008; Becker and Marin, 2009; Kenrick et al., 2012). The streptophyte green algae, also called charophyte algae, inhabit freshwater environments and display a variety of cellular organizations, ranging from unicellular (e.g., *Mesostigma viride*, the only species of the Mesostigmatophyceae and some species of the Zygnematophyceae), to packets of cells (*Chlorokybus atmophyticus*) or filaments (Klebsormidiophyceae and Zygnematophyceae), and to multicellular organization (Coleocheotophyceae and Charophyceae) (Graham et al., 2000; McCourt et al., 2004; Umen, 2014). Identifying which of the six main lineages of streptophyte algae is the closest to land plants proved to be challenging (Karol et al., 2001; Turmel et al., 2006, 2007, 2013; Laurin-Lemay et al., 2012; Zhong et al., 2013); but there is now solid evidence based on both nuclear (Wodniok et al., 2011; Laurin-Lemay et al., 2012; Timme et al., 2012; Zhong et al., 2013; Wickett et al., 2014) and chloroplast phylogenomic studies that the Zygnematophyceae is sister to all land plants (Turmel et al., 2006; Turmel et al., 2007; Civan et al., 2014; Zhong et al., 2014). This morphologically diverse group comprises all green algae that reproduce sexually by conjugation and is the only streptophyte algal class that displays substantial diversity (at least 4000 species) (Gerrath, 2003), with several major lineages identified on the basis of the nuclear-encoded small subunit (SSU) rDNA and chloroplast-encoded *rbcL* gene sequences (Gontcharov et al., 2003, 2004), or on the basis of the chloroplast and mitochondrial *psaA, rbcL* and *cox3* genes (Hall et al., 2008). Based on the structure of the cell wall, the Zygnematophyceae has been divided into two orders: the Zygnematales feature a smooth cell wall (the ancestral trait) and the Desmidiales an ornamented and segmented cell wall (McCourt et al., 2000; Gerrath, 2003). Cell division in the three earliest-diverging lineages of the Streptophyta, the Mesostigmatophyceae, Chlorokybophyceae and Klebsormidiophyceae, occurs by furrowing, but as in land plants, the morphologically more complex Charophyceae and Coleocheotophyceae use a mechanism of cell division involving a phragmoplast and possess cell walls with plasmodesmata (Graham et al., 2000; McCourt et al., 2004).

To date, only nine chloroplast genome sequences of streptophyte algae are available in public databases: four for the Zygnematophyceae and a single genome for each of the remaining streptophyte algal lineages (Lemieux et al., 2000; Turmel et al., 2002, 2005, 2006; Lemieux et al., 2007; Civan et al., 2014). From their gene contents, which vary from 114 to 138 standard genes (i.e., genes whose orthologs are usually found in the chloroplast of photosynthetic eukaryotes), it was predicted that 144 unique standard genes were present in the common ancestor of all streptophytes (Turmel et al., 2006). Comparisons of streptophyte algal genomes with their land plant counterparts revealed that a large fraction of the structural features typically conserved in land plant chloroplast DNAs (cpDNAs) were inherited from streptophyte green algae occupying early diverging lineages (Turmel et al., 2006, 2007; Civan et al., 2014). For instance, 15 of the 21 group II introns found in land plants arose before the emergence of the Klebsormidiophyceae or during the evolutionary interval separating the latter lineage from the Charophyceae (Turmel et al., 2006, 2007; Civan et al., 2014). It was also inferred that the chloroplast genome remained unchanged or mostly unchanged in terms of gene content, gene order, and intron content during the transition from streptophyte algae to land plants (Turmel et al., 2006, 2007). In contrast, extensive gene shuﬄing and substantial structural alterations, including multiple intron losses, took place within the Zygnematophyceae (Turmel et al., 2005; Civan et al., 2014). The highly conserved quadripartite structure of green plant cpDNAs — a structure characterized by two copies of a large inverted repeat (IR) sequence encoding the rRNA operon, which are separated from one another by small and large single-copy (SSC and LSC) regions — was found in only one of the analyzed zygnematophyceans (*Roya anglica*); however, this IR contains a broken rRNA operon and two open reading frames (ORFs) not previously reported in other streptophyte IRs (Civan et al., 2014). This observation led Civan et al. (2014) to propose that either the IR was lost on three independent occurrences during the evolution of the Zygnematophyceae or that it arose *de novo* from an ancestor lacking an IR. In *Klebsormidium flacccidum*, the IR is also unusual with regards to its exceptionally large size and its apparent lack of the 4.5S and 5S rRNA genes from the rRNA operon (Civan et al., 2014).

Sampling of additional streptophyte algal taxa is required to better understand the evolutionary history of the chloroplast genome in the Zygnematophyceae and its evolutionary dynamics in other classes. Toward these goals, we sequenced the chloroplast genomes of the klebsormidiophyceans *Entransia fimbriata* and *Klebsormidium* spec. SAG 51.86, the coleochaetophycean *Coleochaete scutata*, and six zygnematophyceans that were selected to represent distinct lineages in the SSU rDNA and *rbcL* phylogenies reported by Gontcharov et al. (2004): *Cylindrocystis brebissonii, Netrium digitus, Roya obtusa* and *Spirogyra maxima* belong to the Zygnematales, while *Cosmarium botrytis* and *Closterium baillyanum* represent the Desmidiales. Our comparative analyses of these genomes with their previously described streptophyte green algal counterparts indicate that the large IR was involved in major structural changes (IR losses or expansion/contraction) in all three sampled classes and that genes underwent extensive shuﬄing in both the Klebsormidiophyceae and the Zygnematophyceae.

## Materials and Methods

### Strains and Culture Conditions

Strains of *Klebsormidium* sp. SAG 51.86, *Coleochaete scutata* SAG 110.80M, *Closterium baillyanum* SAG 50.89, *Cylindrocystis brebissonii* SAG 615-1, and *Roya obtusa* SAG 168.80 were obtained from the culture collection of algae at the University of Goettingen^1^, whereas *Entransia fimbriata* UTEX LB 2353, *Cosmarium botrytis* UTEX 175, *Netrium digitus* UTEX LB 561, and *Spirogyra maxima* UTEX LB 2495 originated from the culture collection of algae at the University of Texas in Austin^2^. All strains were grown in medium C (Andersen, 2005) at 18°C under alternating 12-h light/dark periods.

### Genome Assemblies and Sequence Analyses

For all strains, except *Cosmarium* and *Cylindrocystis*, total cellular DNA was extracted as described (Turmel et al., 1999a) and A + T-rich organellar DNA was separated from nuclear DNA by CsCl-bisbenzimide isopycnic centrifugation (Lemieux et al., 2014). Total cellular DNA from *Cosmarium* and *Cylindrocystis* was isolated using the EZNA HP Plant Mini Kit of Omega Bio-Tek (Norcross, GA, USA).

For Illumina sequencing of the *Closterium, Cosmarium*, and *Cylindrocystis* chloroplast genomes, libraries of 700-bp fragments were constructed using the TrueSeq DNA Sample Prep Kit (Illumina, San Diego, CA, USA) and paired-end reads were generated on the Illumina HiSeq 2000 (100-bp reads) or the MiSeq (300-bp reads) sequencing platforms by the Innovation Centre of McGill University and Génome Québec^3^ and the “Plateforme d’Analyses Génomiques de l’Université Laval^4^,” respectively. Reads were assembled using Ray v2.3.1 (Boisvert et al., 2010) and contigs were visualized, linked and edited using CONSED v22 (Gordon et al., 1998). Contigs of chloroplast origin were identified by BLAST searches against a local database of organelle genomes. Regions spanning gaps in cpDNA assemblies were amplified by polymerase chain reaction (PCR) with primers specific to the flanking sequences. Purified PCR products were sequenced using Sanger chemistry with the PRISM BigDye Terminator Ready Reaction Cycle Sequencing Kit (Applied Biosystems, Foster City, CA, USA) on ABI model 373 or 377 DNA sequencers (Applied Biosystems).

For 454 sequencing of the *Entransia, Netrium, Roya* and *Spirogyra* chloroplast genomes, shotgun libraries (700-bp fragments) of A + T-rich DNA fractions were constructed using the GS-FLX Titanium Rapid Library Preparation Kit of Roche 454 Life Sciences (Branford, CT, USA). Library construction and 454 GS-FLX DNA Titanium pyrosequencing were carried out by the “Plateforme d’Analyses Génomiques de l’Université Laval^5^.” Reads were assembled using Newbler v2.5 (Margulies et al., 2005) with default parameters, and contigs were visualized, linked and edited using CONSED v22 (Gordon et al., 1998). Identification of cpDNA contigs and gap filling were performed as described above for Illumina sequence assemblies.

For Sanger sequencing of the *Klebsormidium* and *Coleochaete* chloroplast genomes, random clone libraries were prepared from 1500 to 2000-bp fragments derived from A + T rich DNA fractions using the pSMART-HCKan (Lucigen Corporation, Middleton, WI, USA) plasmid. Positive clones were selected by hybridization of each plasmid library with the original DNA used for cloning. DNA templates were amplified using the Illustra TempliPhi Amplification Kit (GE Healthcare, Baie d’Urfé, Canada) and sequenced with the PRISM BigDye terminator cycle sequencing ready reaction kit (Applied Biosystems) on ABI model 373 or 377 DNA sequencers (Applied Biosystems), using SR2 and SL1 primers as well as oligonucleotides complementary to internal regions of the plasmid DNA inserts. The resulting sequences were edited and assembled using Sequencher v5.1 (Gene Codes Corporation, Ann Arbor, MI, USA). Genomic regions not represented in the sequence assemblies or plasmid clones were directly sequenced from PCR-amplified fragments using primers specific to the flanking contigs.

Genes and ORFs were identified on the final assemblies using a custom-built suite of bioinformatics tools as described previously (Turmel et al., 2006). tRNA genes were localized using tRNAscan-SE v1.3.1 (Lowe and Eddy, 1997). Intron boundaries were determined by modeling intron secondary structures (Michel et al., 1989; Michel and Westhof, 1990) and by comparing intron-containing genes with intronless homologs. Circular genome maps were drawn with OGDraw v1.2 (Lohse et al., 2007). Genome-scale sequence comparisons of the pairs of *Roya* and *Klebsormidium* species were carried out with LAST v7.1.4 (Frith et al., 2010). For all compared genomes, G + C contents of a set of 88 protein-coding genes were determined at the three codon positions using DAMBE v5 (Xia, 2013).

To estimate the proportion of small repeated sequences, repeats ≥ 30 bp were retrieved using REPFIND of the REPuter v2.74 program (Kurtz et al., 2001) with the options -f -p -l -allmax and were then masked on the genome sequences using RepeatMasker^6^ running under the Crossmatch search engine^7^. The G+C contents of the repeated and unique sequences were calculated from the outputs of RepeatMasker that were generated with the -xsmall option (under this option the repeat regions are returned in lower case and non-repetitive regions in capitals in the masked file).

### Phylogenetic Analyses

The chloroplast genomes of 28 streptophyte taxa were used to generate the analyzed amino acid (PCG-AA) and nucleotide (PCG12) data sets. The latter were assembled from the following 88 protein-coding genes: *accD, atpA, B, E, F, H, I, ccsA, cemA, chlB, I, L, N, clpP, ftsH, infA, ndhA, B, C, D, E, F, G, H, I, J, K, odpB, petA, B, D, G, L, N, psaA, B, C, I, J, M, psbA, B, C, D, E, F, H, I, J, K, L, M, N, T, Z, rbcL, rpl2, 14, 16, 20, 21, 22, 23, 32, 33, 36, rpoA, B, C1, C2, rps2, 3, 4, 7, 8, 11, 12, 14, 15, 16, 18, 19, ycf1, 3, 4, 12, 62, 66.*

The PCG-AA data set was prepared as follows: the deduced amino acid sequences from the 88 individual genes were aligned using MUSCLE v3.7 (Edgar, 2004), the ambiguously aligned regions in each alignment were removed using TrimAl v1.3 (Capella-Gutierrez et al., 2009) with the options block = 6, gt = 0.7, st = 0.005 and sw = 3, and the protein alignments were concatenated using Phyutility v2.2.6 (Smith and Dunn, 2008). Phylogenies were inferred from the PCG-AA data set using the maximum likelihood (ML) and Bayesian methods. ML analyses were carried out using RAxML v8.2.3 (Stamatakis, 2014) and the GTR + Γ4 model of sequence evolution; in these analyses, the data set was partitioned by gene, with the model applied to each partition. Confidence of branch points was estimated by fast-bootstrap analysis (f = a) with 100 replicates. Bayesian analyses were performed with PhyloBayes v4.1 (Lartillot et al., 2009) using the site-heterogeneous CATGTR + Γ4 model (Lartillot and Philippe, 2004). Five independent chains were run for 2,000 cycles and consensus topologies were calculated from the saved trees using the BPCOMP program of PhyloBayes after a burn-in of 500 cycles. Under these conditions, the largest discrepancy observed across all bipartitions in the consensus topologies (maxdiff) was 0.0007, indicating that convergence between the chains was achieved.

The PCG12 nucleotide data set (first and second codon positions) was prepared as follows. The multiple sequence alignment of each protein was converted into a codon alignment, the poorly aligned and divergent regions in each codon alignment were excluded using Gblocks v0.91b (Castresana, 2000) with the -*t* = c, -*b*3 = 5, -*b*4 = 5 and -*b*5 = half options, and the individual gene alignments were concatenated using Phyutility v2.2.6 (Smith and Dunn, 2008). The third codon positions of the resulting PCG123 alignment were then excluded using Mesquite v3.04 (Maddison and Maddison, 2015) to produce the PCG12 data set. ML analysis of the PCG12 data set was carried out using RAxML v8.2.3 (Stamatakis, 2014) and the GTR + Γ4 model of sequence evolution. This data set was partitioned into gene groups, with the model applied to each partition. Confidence of branch points was estimated by fast-bootstrap analysis (f = a) with 100 replicates.

d*N*, d*S* and d*N/*d*S* trees were inferred from a *tufA* codon alignment prepared as described above using PAML v4.8a (Yang, 2007) and the F3X4 codon frequencies model implemented in codeml. Positive selection was tested across the *tufA* sequences using the PARRIS module implemented in Datamonkey (Delport et al., 2010).

### Analyses of Gene Order and Reconstruction of Genomic Character Evolution

Syntenic regions in pairwise genome comparisons were identified using a custom-built program and the number of gene reversals between the compared genomes was estimated with GRIMM v2.01 (Tesler, 2002). The same custom-built program was employed to convert gene order to all possible pairs of signed genes (i.e., taking into account gene polarity); the gene pairs conserved in three or more genomes were visualized using Mesquite v3.04 (Maddison and Maddison, 2015). Gains and/or losses of genomic characters (standard genes, introns and gene pairs) were mapped on the streptophyte tree topology inferred in this study using MacClade v4.08 (Maddison and Maddison, 2000) and the Dollo principle of parsimony.

A ML tree based on gene adjacency was inferred using the phylogeny reconstruction option of the MLGO web server (Hu et al., 2014) and a gene order matrix containing all standard genes (including all copies of duplicated genes). Confidence of branch points was estimated by 1000 bootstrap replications. A gene reversal tree with the same topology as the MLGO tree was also computed; branch lengths were estimated using the -t option of MGR v2.03 (Bourque and Pevzner, 2002) and a gene order matrix of the 89 genes shared by all compared genomes; because MGR cannot handle duplicated genes, only one copy of the IR and of each duplicated gene was included in this analysis.

### Availability of Supporting Data

The chloroplast genome sequences generated in this study are available in the GenBank database under the accession numbers KU646489-KU646497. The data sets supporting the results of this article are available in the TreeBASE repository (Study ID 19085).

## Results

### General Features

**FIGURE 1:**
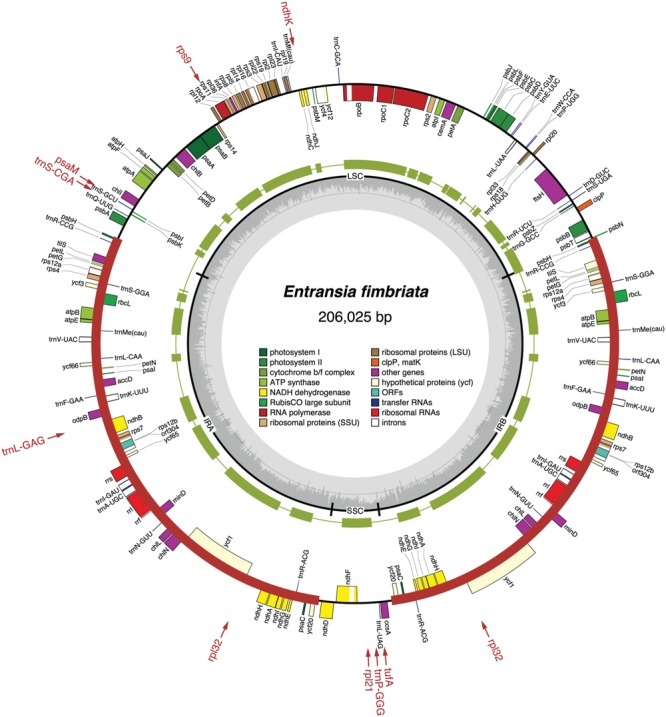
**Gene map of the *Entransia* chloroplast genome.** Filled boxes represent genes, with colors denoting gene categories as indicated in the legend; the red thick lines denote the positions of the IR sequences. Genes on the outside of each map are transcribed counterclockwise; those on the inside are transcribed clockwise. The green thick lines in the second outermost middle ring represent the gene clusters conserved between the *Entransia* and *Klebsormidium flaccidum* cpDNAs. The genes shown in red are present in *Klebsormidium* but are missing in *Entransia*; the arrows associated with these genes point to the cpDNA regions where they are located in *Klebsormidium*. The innermost ring indicates the positions of the IR, SSC, and LSC regions, and the gray histogram represents the G + C percentages calculated with OGDRAW (light gray, A + T; dark gray, G + C).

**FIGURE 2:**
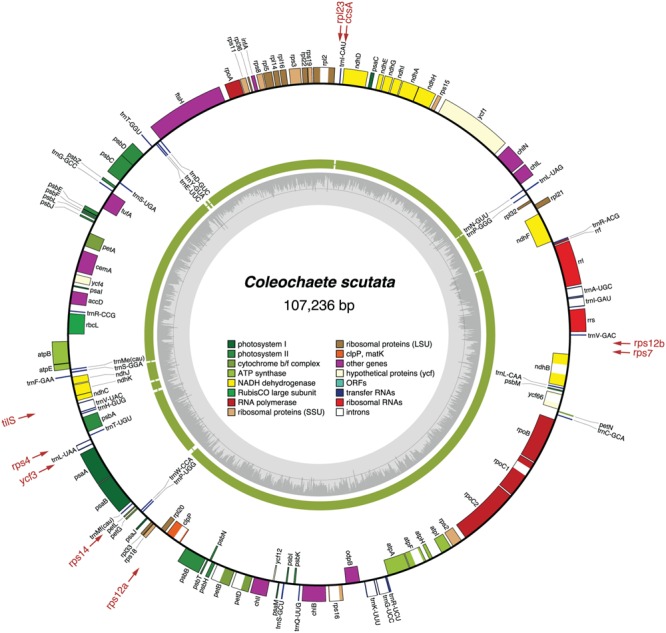
**Gene map of the *Coleochaete* chloroplast genome.** Filled boxes represent genes, with colors denoting gene categories as indicated in the legend. Genes on the outside of each map are transcribed counterclockwise; those on the inside are transcribed clockwise. The green thick lines in the second outermost middle ring represent the gene clusters conserved between the *Coleochaete* and *Chaetosphaeridium* cpDNAs. The genes shown in red are present in *Chaetosphaeridium* but are missing in *Coleochaete*; the arrows associated with these genes point to the cpDNA regions where they are located in *Chaetosphaeridium*. The gray histogram on the inside of each map represents the G + C percentages calculated with OGDRAW (light gray, A + T; dark gray, G + C).

**FIGURE 3:**
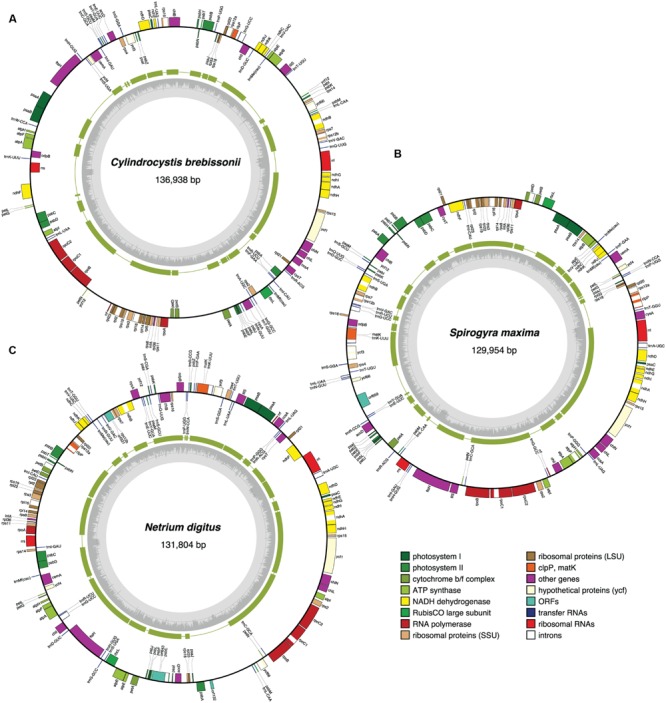
**Gene maps of the *Cylindrocystis***(A)**, *Spirogyra***(B)**, and *Netrium***(C)** chloroplast genomes.** Filled boxes represent genes, with colors denoting gene categories as indicated in the legend. Genes on the outside of each map are transcribed counterclockwise; those on the inside are transcribed clockwise. The green thick lines in the second outermost middle rings represent the gene clusters conserved between the *Cylindrocystis* and *Zygnema* cpDNAs **(A)**, the *Spirogyra* and *Netrium* cpDNAs **(B)**, and the *Netrium* and *Roya* cpDNAs **(C)**. The gray histogram on the inside of each map represents the G + C percentages calculated with OGDRAW (light gray, A + T; dark gray, G + C).

**FIGURE 4:**
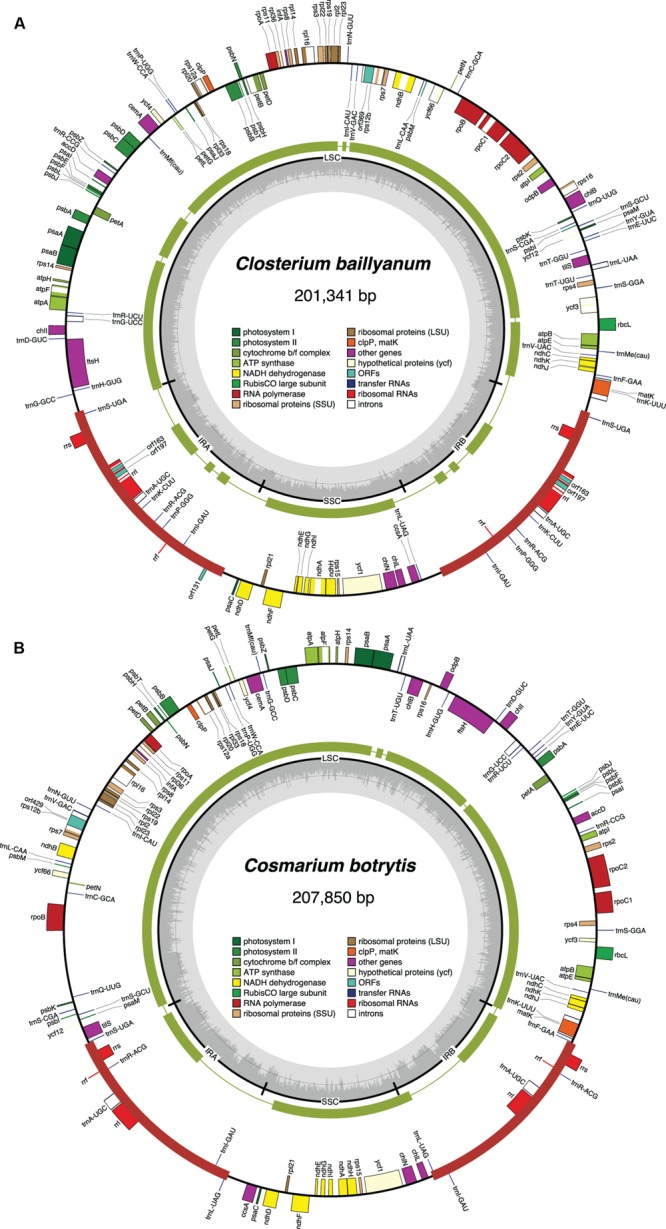
**Gene maps of the *Closterium***(A)** and *Cosmarium***(B)** chloroplast genomes.** Filled boxes represent genes, with colors denoting gene categories as indicated in the legend; the red thick lines denote the positions of the IR sequences. Genes on the outside of each map are transcribed counterclockwise; those on the inside are transcribed clockwise. The green thick lines in the second outermost middle rings represent the gene clusters conserved between the *Closterium* and *Roya* cpDNAs **(A)** and the *Cosmarium* and *Staurastrum* cpDNAs **(B)**. The innermost ring indicates the positions of the IR, SSC and LSC regions. The innermost ring indicates the positions of the IR, SSC and LSC regions, and the gray histogram represents the G + C percentages calculated with OGDRAW (light gray, A + T; dark gray, G + C).

**FIGURE 5:**
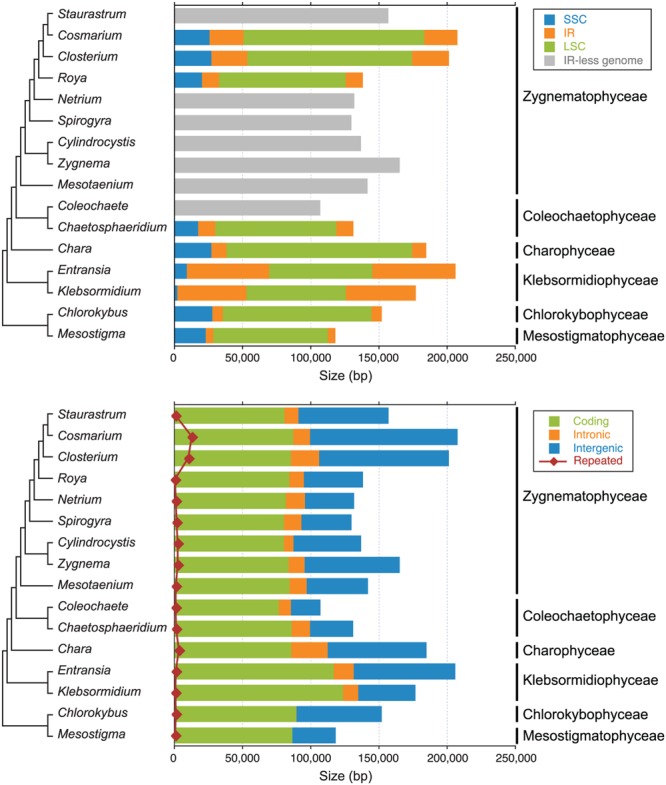
**Sizes of genomic regions and different types of sequences in the streptophyte chloroplast genomes compared in this study.** The sizes of the SSC, IR and LSC regions are shown in the upper panel, whereas the sizes of coding, intronic, intergenic and small repeated sequences (≥30 bp) are shown in the lower panel. Note that intron-encoded genes were not considered as coding sequences but rather as intron sequences. The phylogenetic relationships among the taxa examined are also presented (see **Figure 6** for details).

The chloroplast genomes of the nine sampled taxa, except that of *Klebsormidium* sp. SAG 51.86, were entirely sequenced and assembled as circular-mapping molecules (**Figures 1–4**). Although these genomes assembled as circles, it is possible that they exist *in vivo* as multi-genomic, linear-branched structures, as reported for land plant cpDNAs (Bendich, 2004). Their sizes range from 107 (for *Coleochaete*) to 208 kb (for *Cosmarium*) (**Figures 1–4**). Genome size variation is also important among lineages of the same class, in particular within the Zygnematophyceae where the variation is 1.6-fold (from 130 kb in *Spirogyra* to 208 kb in *Cosmarium*). Within the Klebsormidiophyceae, differences in IR size, intron content, and lengths of intergenic regions essentially account for the increased size of the *Entransia* genome (206 kb) compared to that of *Klebsormidium flaccidum* (177 kb) (**Table 1**; **Figure 5**). Note that we detected an IR in the partially sequenced *Klebsormidium* sp. SAG 51.86 genome but were unable to identify the IR/SSC junction; 91% of this 130,962-kb genome sequence could be aligned with the *Klebsormidium flaccidum* genome in a strictly colinear fashion, highlighting the absence of just two genes [*trnN*(guu) and *ccsA*] and a total of 7,551 divergent positions, i.e., 6.3% of the aligned sequence. Within the Coleochaetophyceae, the 24-kb difference between the *Chaetosphaeridium* and *Coleochaete* genomes is largely due to the absence of the IR and of eight genes, as well as to shorter intergenic regions in *Coleochaete* (**Table 1**; **Figure 5**). For the Zygnematophyceae, the observed genome size variation is attributable to combinations of all the abovementioned factors (**Table 1**; **Figure 5**). Only half of the six newly sequenced zygnematophycean genomes display an IR: the 12.6-kb IR of *Roya obtusa* is about two-fold smaller than those of *Closterium* and *Cosmarium*, but is about the same size as that found in the coleochaetophycean *Chaetosphaeridium*. The newly sequenced zygnematophycean genomes also differ greatly in intron content. For instance, the *Closterium* and *Cosmarium* genomes, which are the largest among the examined zygnematophyceans, display the lowest and highest numbers of introns, respectively; moreover, they exhibit the highest proportion of repeats ≥ 30 bp (**Table 1**). Note that the alignment of the *Roya obtusa* and *Roya anglica* cpDNAs revealed that these genomes are colinear over their entire length and that their sequences diverge at only 74 sites (i.e., 0.05% divergence), 12 of which are indels of 1, 2, or 4 nucleotides.

**Table 1:** General features of the streptophyte green algal chloroplast genomes compared in this study.

Taxon	Accession no.^a^	Size (bp)				G+C (%)	Genes		Introns (no.)		Repeats^b^ (%)
		Total	IR	LSC	SSC		No.^c^	% genome^d^	Group I	Group II^e^	
Mesostigmatophyceae											
*Mesostigma viride* NIES 296	NC_002186	118,360	6,057	83,627	22,619	30.1	137	73.2			0.2
Chlorokybophyceae											
*Chlorokybus atmophyticus* SAG 48.80	NC_008822	152,254	7,640	109,098	27,876	38.2	138	58.8	1		0.5
Klebsormidiophyceae											
*Klebsormidium* sp. SAG 51.86^f^	KU646497^∗^	>130,962^g^				40.6	112	67.7		12 (2)	0
*Klebsormidium flaccidum* SAG 121.8	NC_024167	176,832	51,118	72,779	1,817	42.0	114	76.3		12 (2)	0.2
*Entransia fimbriata* UTEX LB 2353	KU646490^∗^	206,025	60,590	75,629	9,216	33.0	118	64.4	1	10 (1)	0.4
Charophyceae											
*Chara vulgaris*	NC_008097	184,933	10,919	135,815	27,280	26.2	127	60.9	2	16 (1)	1.6
Coleochaetophyceae											
*Chaetosphaeridium globosum* M1311	NC_004115	131,183	12,431	88,682	17,639	29.6	125	76.9	1	17 (1)	0.7
*Coleochaete scutata* SAG 110.80M	KU646493^∗^	107,236				27.6	117	79.9	1	14 (0)	0.6
Zygnematophyceae											
*Mesotaenium endlicherianum* SAG 12.97	NC_024169	142,017				42.2	124	68.4	1	16 (1)	0.6
*Zygnema circumcarinatum* SAG 698-1a	NC_008117	165,372				31.1	125	57.8	1	12 (1)	1.2
*Cylindrocystis brebissonii* SAG 615-1	KU646495^∗^	136,938				29.8	122	63.8	1	9 (1)	1.5
*Spirogyra maxima* UTEX LB 2495	KU646489^∗^	129,954				30.1	124	73.3	1	16 (1)	1.0
*Netrium digitus* UTEX LB 561	KU646491^∗^	131,804				31.4	125	74.5	1	13 (1)	0.5
*Roya anglica* ACOI 799	NC_024168	138,275	12,568	92,926	20,213	33.1	122	69.7	1	9 (1)	0.1
*Roya obtusa* SAG 168.80	KU646496^∗^	138,272	12,568	92,924	20,212	33.1	122	69.9	1	9 (1)	0.1
*Closterium baillyanum* SAG 50.89	KU646494^∗^	201,341	26,784	120,746	27,027	39.3	124	53.1	4	15 (1)	4.9
*Cosmarium botrytis* UTEX 175	KU646492^∗^	207,850	24,465	132,863	26,057	39.8	122	47.9	1	6 (1)	6.0
*Staurastrum punctulatum* SAG 679-1	NC_008116	157,089				32.5	122^h^	58.4	1	7 (1)	0.3

*^a^The asterisks denote the nine genomes described here for the first time. ^b^Non-overlapping repeat elements were mapped on each genome with RepeatMasker using the repeats ≥ 30 bp identified with REPuter as input sequences. ^c^Only standard genes conserved in green plant chloroplast genomes are included in these values. Duplicated genes were counted only once. ^d^These values include the sequences of standard genes and ORFs coding for proteins of known functions. ^e^The first value provides the total number of group II introns; the number of *trans*-spliced introns is given in parentheses. ^f^This species was formally called *Microspora stagnorum* (Mikhailyuk et al., 2008). ^g^The exact size of the *Klebsormidium* sp. SAG 51.86 genome could not be determined because the IR/SSC junction was not identified. The features of this genome were estimated from the partial sequence. ^h^This value includes the *trnS*(cga) pseudogene found in the *Staurastrum* genome (Turmel et al., 2005).*

G + C content at the genome level varies from 26.2 to 42.2%, and at the high end of this range are found the cpDNAs of *Chlorokybus, Klebsormidium flaccidum* and of the zygnematophyceans *Mesotaenium, Closterium* and *Cosmarium* (**Table 1**). Although coding regions have generally a higher G + C content than introns and intergeneric regions, the *Cosmarium* genome harbors an excess of guanines and cytosines in its intergenic regions compared to the rest of the genome sequence (Supplementary Figure S1A). The *Klebsormidium flaccidum* and *Mesotaenium* coding regions display the highest G + C content at the first, second and third codon positions (Supplementary Figure S1B). The dispersed repeats present in both the *Closterium* and *Cosmarium* genomes are richer in G + C than unique sequences (52.5 and 60.7% versus 38.6 and 38.5%, respectively).

### Phylogenomic Analyses

**FIGURE 6:**
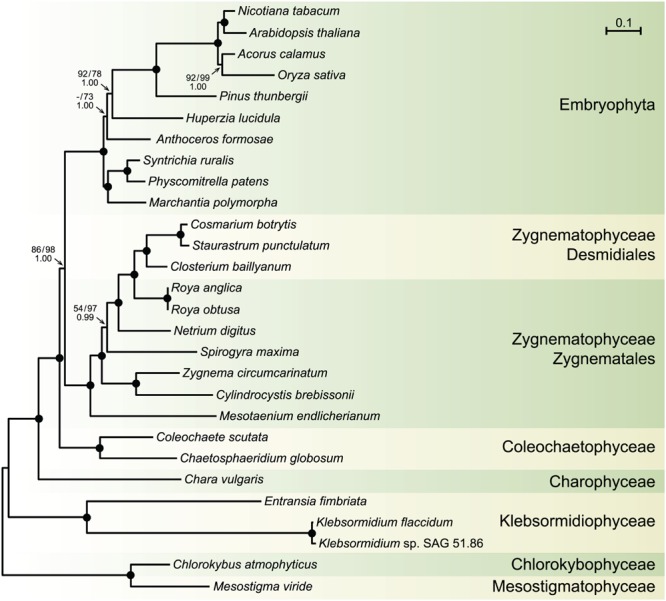
**Phylogeny of streptophytes inferred using amino acid and nucleotide data sets assembled from 88 chloroplast protein-coding genes.** The best-scoring tree inferred with Phylobayes from the PCG-AA data set under the CATGTR + Γ4 model is presented. *Mesostigma* and *Chlorokybus* were used as outgroup. Bootstrap support (BS) and posterior probability (PP) values are reported on the nodes: on top from left to right, are shown the BS values for the RAxML analyses of the PCG-AA and PCG12 data sets, respectively; the PP values of the Phylobayes CATGTR + Γ4 analysis are presented under the BS values. A black dot indicates that the corresponding branch received maximum support in all three analyses; a dash represents a BS value <50%. The scale bar denotes the estimated number of amino acid substitutions per site. The GenBank accession numbers of the streptophyte algal chloroplast genomes included in the PCG-AA and PCG12 data sets are provided in **Table 1**. The GenBank accession numbers of the land plant chloroplast genomes are as follows: *Marchantia polymorpha* NC_001319, *Physcomitrella patens* NC_005087, *Syntrichia ruralis* NC_012052, *Anthoceros formosae* NC_004543, *Huperzia lucidula* NC_006861, *Pinus thunbergii* NC_001631, *Oryza sativa* NC_001320, *Acorus calamus* NC_007407, *Arabidopsis thaliana* NC_000932, *Nicotiana tabacum* NC_001879.

Before moving on to other comparative genome analyses, we provide here the phylogenetic context that will be necessary to interpret the results of these analyses. We analyzed an amino acid data set (PCG-AA, 18,646 sites) and a nucleotide data set (PCG12, 38,354 sites), both derived from the same set of 88 chloroplast protein-coding genes from 28 streptophytes — 18 algae and ten selected land plants — using the Bayesian and/or ML methods (**Figure 6**). Missing data account for only 5.2% of each data set. Regardless of the data set or the reconstruction method used, identical relationships were recovered for the streptophyte algae, with the Zygnematophyceae being sister to land plants. In the latter algal class, *Mesotaenium* represents the earliest-diverging lineage and is followed by the clade formed by *Zygnema* and *Cylindrocystis*, and next by the *Spirogyra* and *Netrium* lineages. The two *Roya* species, which also belong to the Zygnematales order, are sister to the desmidialean clade uniting *Closterium* with *Cosmarium* and *Staurastrum*. All the nodes associated with the streptophyte algal lineages received strong statistical support in the ML protein tree, except the inner node subtending the branch leading to *Spirogyra* and its sister lineages. The protein and gene phylogenies differed only with respect to the branching order of the bryophyte lineages.

### Gene Content

#### Standard Genes

**FIGURE 7:**
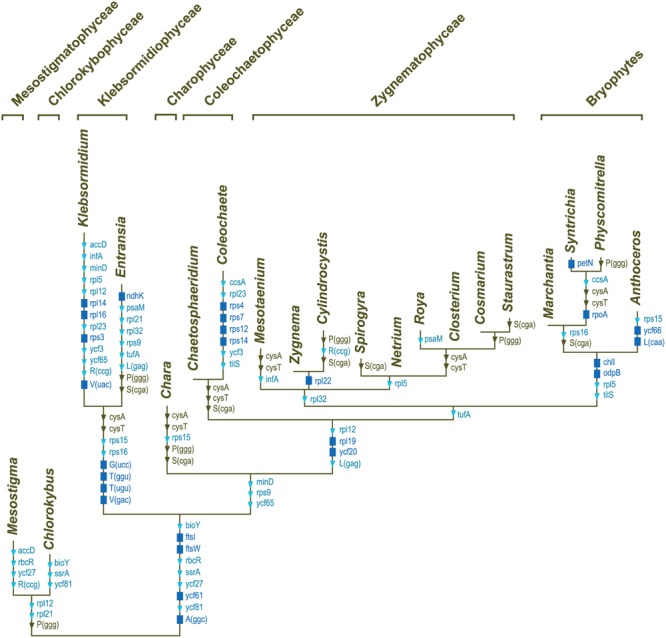
**Mapping of gene losses on the streptophyte phylogeny inferred in this study.** Of the 144 genes that were present in the common ancestor of the 20 compared streptophytes, 90 were retained in all taxa: *atpA, B, E, F, H, I, cemA, chlB, L, N, clpP, ftsH, ndhA, B, C, D, E, F, G. H, I, J, petA, B, D, G, L, psaA, B, C, I, J, psbA, B, C, D, E, F, H, I, J, K, L, M, N, T, Z, rbcL, rpl2, 20, 33, 36, rpoB, C1, C2, rps2, 8, 11, 18, 19, rrf, rrl, rrs, ycf1, 4, 12, trnA*(ugc), *C*(gca), *D*(guc), *E*(uuc), *F*(gaa), *G*(gcc), *H*(gug), *I*(cau), *I*(gau), *K*(uuu), *L*(uaa), *L*(uag), *Me*(cau), *Mf*(cau), *N*(guu), *P*(ugg), *Q*(uug), *R*(acg), *R*(ucu), *S*(gcu), *S*(gga), *S*(uga), *W*(cca), *Y*(gua). The genes denoted by the dark blue rectangles represent synapomorphic losses, while the genes denoted by the triangles indicate homoplasic losses (light blue symbols, ≤3 independent losses; brown symbols, more than three independent losses).

All compared genomes share a set of 90 genes coding for three rRNAs (*rrs, rrl* and *rrf*), 24 tRNAs, and 63 proteins (see legend of **Figure 7**). Therefore, of the 144 standard genes predicted to have been present in the common ancestor of all streptophytes, 54 (42 protein-coding genes, 11 tRNA genes and the *ssrA* gene coding for tmRNA) experienced losses from the chloroplast during diversification of streptophyte algae (**Figure 7**). A total of 103 gene losses was inferred by mapping these genes on the phylogeny shown in **Figure 6**. Only 26 of these events occurred once, many of which represent signatures uniting different streptophyte classes or divergent lineages belonging to the same class.

Subsequent to the losses of three genes during the evolutionary period preceding the emergence of the common ancestor of *Chaetosphaeridium* and *Coleochaete*, eight genes disappeared in the lineage leading to *Coleochaete*. The reduced number of genes in klebsormidiophyceans is the result of eight gene losses that occurred during the interval leading to the common ancestor of the *Klebsormidium* and *Entransia* genera and of 22 gene losses that occurred in the lineages leading to *Klebsormidium* and *Entransia*. Note that the *rrf* gene encoding the 5S rRNA is not included in the list of *Klebsormidium* losses; Civan et al. (2014) failed to identify this gene in [GenBank:NC_024167] but we discovered its highly divergent sequence at coordinate positions 92980–93102 and 156632–156510 during the course of this study. We also found the 3′ *rrl* sequence that was reported to be missing in the same alga (Civan et al., 2014); this sequence corresponds to the 4.5S rRNA that is part of the land plant large-subunit rRNA. The complete *rrl* gene spans coordinate positions 89958–92857 and 159654–156755 in the *Klebsormidium* genome [GenBank:NC_024167].

Of the 123 genes predicted to have been present in the common ancestor of all land plants, fewer than five are missing in the Zygnematophyceae. *Netrium* is missing *rpl32*, and in addition to the latter gene, *Zygnema* is missing *rpl22* and *Spirogyra trnS*(cga). The *cysA* and *cysT* genes encoding components of the sulfate transport system are found in early-diverging lineages of the Zygnematophyceae (*Zygnema, Cylindrocystis, Spirogyra* and *Netrium*) but are missing in all other streptophyte lineages, except in the basal Mesostigmatophyceae and Chlorokybophyceae.

The gene loss scenario discussed above does not include *trnK*(cuu), a tRNA gene found exclusively in the zygnemataleans *Mesotaenium* and *Closterium*. BlastN similarity searches against the non-redundant database of NCBI indicated that this gene arose from duplication and subsequent sequence divergence of *trnN*(guu). Prior to our study, the trebouxiophycean *Stichococcus bacillaris* was the only known green alga carrying *trnK*(cuu) in its chloroplast; however, it was found to originate from duplication of *trnK*(uuu) (Turmel et al., 2015).

#### Increased Mutation Rates of *tufA* in the Coleochaetophyceae

**FIGURE 8:**
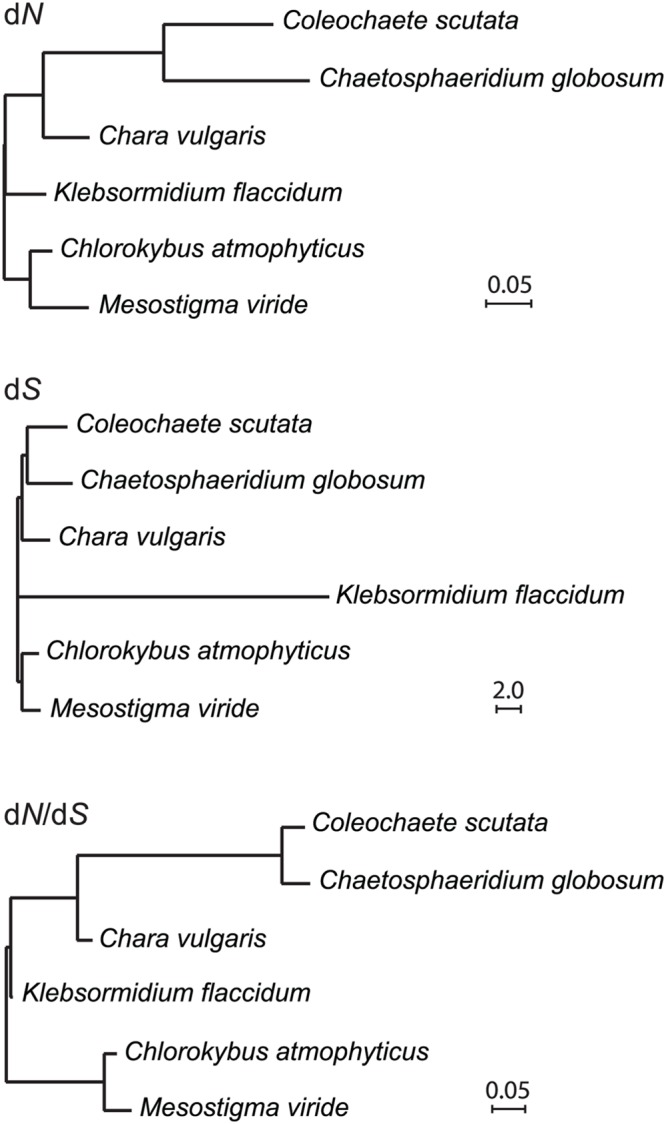
**Accelerated evolution of *tufA* in the Coleochaetophyceae.** d*N*, d*S* and d*N*/d*S* branch lengths were calculated using PAML v4.8a and the streptophyte tree topology inferred in this study.

The *Coleochaete scutata tufA* gene encoding the protein synthesis factor Tu has retained an intact open reading frame but like its *Chaetosphaeridium* and *Coleochaete orbicularis* counterparts, it is highly divergent in sequence compared to the corresponding genes found in *Mesostigma, Chlorokybus* and *Chara*. Our alignment of streptophyte *tufA* sequences also revealed a marked G + C bias at the third codon positions of the *Klebsormidium* sequence. We calculated d*N*, d*S* and d*N*/d*S* branch lengths for *tufA* based on the relationships we inferred in **Figure 6** and found that the d*N* branches subtending and within the *Coleochaete* + *Chaetosphaeridium* clade are very long compared to all other examined streptophyte algae (**Figure 8**). The long branch subtending the *Coleochaete* + *Chaetosphaeridium* clade in the d*N*/d*S* tree might suggest that *tufA* experienced positive selection during early evolution of coleochaetophyceans. However, no evidence for positive selection across the *tufA* sequence (*P* < 0.1) was obtained in the likelihood ratio test implemented in the PARRIS module of Datamonkey (Delport et al., 2010).

#### Non-standard Genes

Freestanding ORFs showing similarities (*E*-value threshold of 1e-08) with recognized protein domains were identified in zygnematophycean chloroplast genomes (**Table 2**). They encode type II intron maturases (MatK), a phage DNA primase and DNA breaking-rejoining enzymes (recombinase/integrase).

**Table 2:** Non-standard genes identified as freestanding ORFs in the chloroplast genomes compared in this study.

Taxon	ORF^a^	Coordinates^b^	Conserved domain
*Mesotaenium endlicherianum*	519	108900–110459	Type II intron maturase MatK (CHL00002)
*Zygnema circumcarinatum*	531	66206–67801	Type II intron maturase MatK (CHL00002)
*Spirogyra maxima*	659	72632–74611	Phage integrase family (pfam00589) and integrase core domain (pfam00665)
*Netrium digitus*	132	102944–103342	Phage integrase family (pfam00589)
*Roya obtusa / Roya anglica*	268	96776–97582	Phage integrase family (pfam00589)
*Staurastrum punctulatum*	108	142226–142552	Phage DNA primase, D5 N terminal like domain (pfam08706)

*^a^Reported here are the freestanding ORFs larger than 100 codons that revealed similarity (*E*-value threshold of 1e-08) with recognized protein domains in BlastP searches. Each ORF is identified by the number of amino acid residues in the encoded protein. ^b^Genomic coordinates of the ORFs in the GenBank accessions provided in **Table 1**.*

The *Roya obtusa* genome contains an ORF (*orf230*, coordinates 102713-103405 in GenBank:KU646496) that is similar to chloroplast ORFs present in *Roya anglica* and two ferns of the Ophioglossaceae, *Mankyua chejuensis* and *Ophioglossum californicum*. In addition, the *psbJ*-*petA* intergenic regions of *Netrium, Roya obtusa, Closterium*, and *Cosmarium* display an ORF encoding a hypothetical protein resembling those encoded at the same genomic locations in *Zygnema, Roya anglica*, and *Staurastrum*. The alignment of these hypothetical proteins indicates that the N-terminal portion is the most conserved (Supplementary Figure S2); however, given the small size and close proximity of this region to the 3′ end of *petA*, the corresponding DNA sequence possibly represents a conserved regulatory sequence.

In *Klebsormidium* sp. SAG 51.86 cpDNA, *orf453* encodes a protein with reverse transcriptase (RT) and intron maturase domains that is highly similar to a freestanding ORF annotated as *matK* in the corresponding sequence of the *Klebsormidium flaccidum* genome. This coding sequence is more likely to be part of the group II intron fragment linked with *psbA* exon 1, and consistent with this view, it shows less similarity to the *matK* genes encoded by *trnK*(uuu) introns than with the ORFs encoded by other group II introns (e.g., those in *Pyramimonas parkeae atpB, Tydemania expeditionis psbC* and *Jenufa minuta psbB*).

### Intron Distribution

**FIGURE 9:**
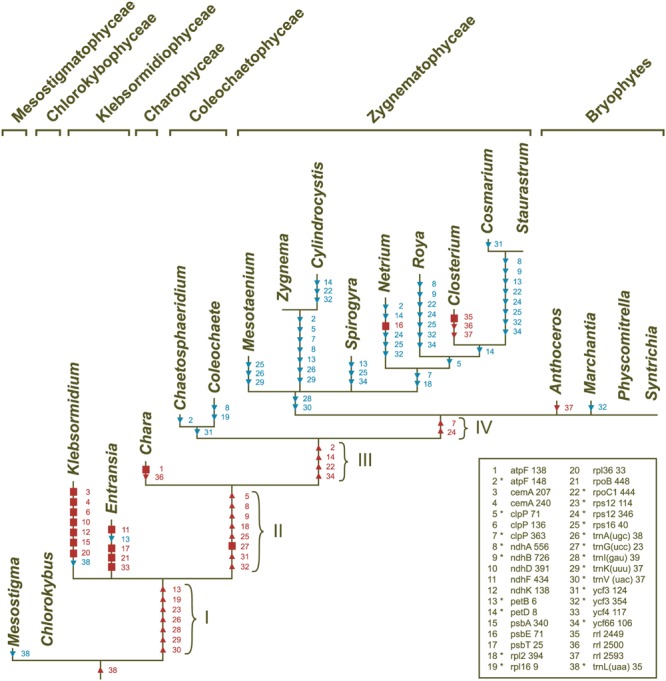
**Gains and losses of chloroplast introns during streptophyte evolution.** These events were inferred using MacClade v4.08 and the streptophyte topology shown in **Figure 6**. The introns denoted by squares represent synapomorphic gains, while those denoted by triangles represent homoplasic gains (red symbols) and losses (blue symbols). Intron losses resulting from losses of the host genes are not shown. The legend indicates the correspondence between character numbers and intron sites; characters 1–34 and 35–38 denote group II and group I introns, respectively. The group II introns shared between streptophyte algae and land plants (those denoted by asterisks) were acquired along the four branches identified by roman numerals.

Introns in streptophyte algal cpDNAs are inserted at 38 distinct sites, 22 of which are shared with most land plants [21 group II intron sites and the *trnL*(uaa)_35 group I intron site]. Only four sites hold group I introns (*rrl*_2449, *rrl*_2500, *rrl*_2593, and *trnL*(uaa)_35). **Figure 9** shows the scenario of chloroplast intron gains and losses that we reconstructed by mapping the presence/absence of introns on the phylogeny inferred in this study. All streptophyte algal genomes, except *Mesostigma* and *Klebsormidium* cpDNAs, contain the *trnL*(uaa)_35 intron. Group II introns shared with land plants were acquired during four distinct evolutionary intervals (these branches are denoted by roman numerals in **Figure 9**), but most of these introns were later lost on one or more occasions. Within the Zygnematophyceae, 43 events of intron losses involving 18 sites were recorded; only the *trans*-spliced *rps12*_114 and the *cis*-spliced *rpl16*_9 and *trnG*(ucc)_23 introns were spared from losses. The *trnI*(gau)_39 and *trnV*(uac)_37 introns were lost before the emergence of this clade.

The only ORF-containing group II introns that streptophyte algae share with land plants are the *trnK*(uuu)_37 and *trans*-spliced *rps12*_114 introns, but the ORF is not conserved in all algal taxa carrying these introns. The ORF encoding the intron maturase MatK is missing from the *trnK*(uuu)_37 introns of *Coleochaete* and the Klebsormidiophyceae, and there is no *trnK*(uuu)_37 intron in the early diverging zygnematophyceans *Mesotaenium, Cylindrocystis* and *Zygnema*; however, the *matK* ORF is freestanding in both *Mesotaenium* and *Zygnema* (**Table 2**). An ORF is also absent from the *trans*-spliced *rsp12*_114 intron in *Klebsormidium, Chara, Spirogyra*, and the three zygnematophyceans just mentioned.

The group II introns that are not shared with land plants occur in *Klebsormidium* (sites 3, 4, 6, 10, 12, 15, and 20), *Entransia* (sites 11, 17, 21, and 33), *Chara* (site 1) and *Netrium* (site 16) (**Figure 9**). Only two of these lineage-specific introns (the *Klebsormidium psbA* and *Netrium psbE* introns) harbor an ORF coding for a putative protein with RT and/or maturase domains. The group I introns that are not shared with land plants are found in *Chara* (site 36) and *Closterium* (sites 35, 36, and 37).

### Gene Organization

**FIGURE 10:**
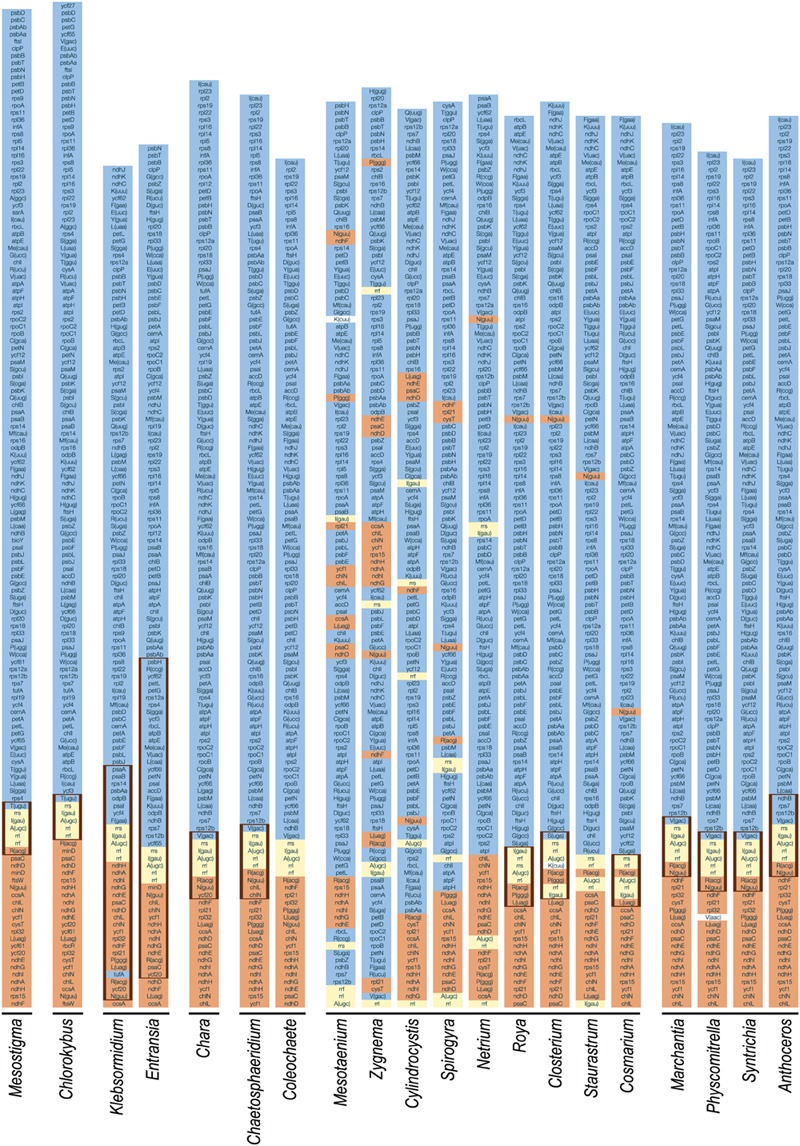
**Gene partitioning patterns of streptophyte chloroplast genomes.** For each genome, one copy of the IR (thick vertical lines) and the entire SSC and LSC regions are represented. The five genes composing the rDNA operon are highlighted in yellow. The color assigned to each of the remaining genes is dependent upon the position of the corresponding gene relative to the rDNA operon in the cpDNA of the streptophyte alga *Mesostigma viride*, a genome displaying an ancestral gene partitioning pattern. The genes highlighted in orange are found within or near the SSC region in this streptophyte genome (downstream of the rDNA operon), whereas those highlighted in blue are found within or near the LSC region (upstream of the rDNA operon). To simplify the comparison of gene order, some genomes are represented in their alternative isomeric forms relative to the genome sequences deposited in GenBank. Note that the *trnK*(cuu) genes of *Mesotaenium* and *Closterium*, and the *trnV*(aac) gene of *Physcomitrella* were not color-coded because their evolutionary origins are unclear.

**FIGURE 11:**
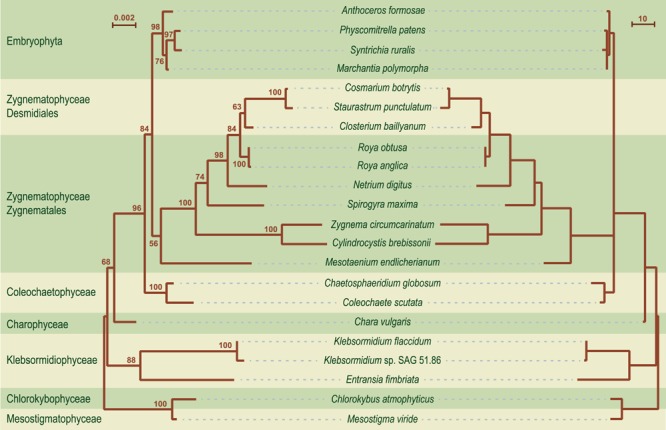
**Phylogenetic relationships of streptophytes based on chloroplast gene order.** On the left is shown the tree inferred using the MLGO web server and a data set containing all standard genes in each genome (including both copies of duplicated genes); BS values are reported on the nodes. The scale bar indicates the number of changes in gene adjacency per site. The tree on the right displays the branch lengths estimated using the user tree (-t) option of MGR v2.03. The scale bar corresponds to the estimated number of gene reversals.

**FIGURE 12:**
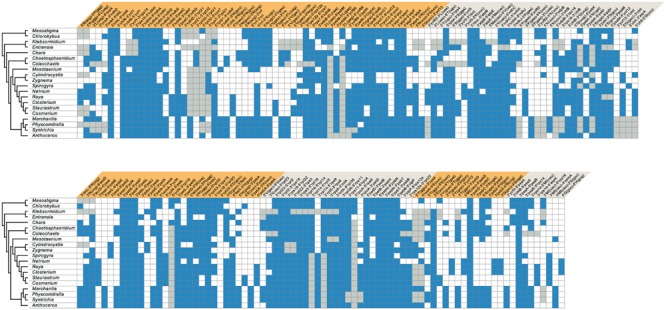
**Shared gene pairs in streptophyte chloroplast genomes.** The gene pairs shared by at least three taxa were identified among all possible signed gene pairs in the compared genomes. The presence of a gene pair is denoted by a blue box; a gray box refers to a gene pair in which at least one gene is missing due to gene loss. Gene pairs are organized in blocks of contiguous gene pairs (shown as alternating colors) to facilitate the identification of conserved gene clusters.

**FIGURE 13:**
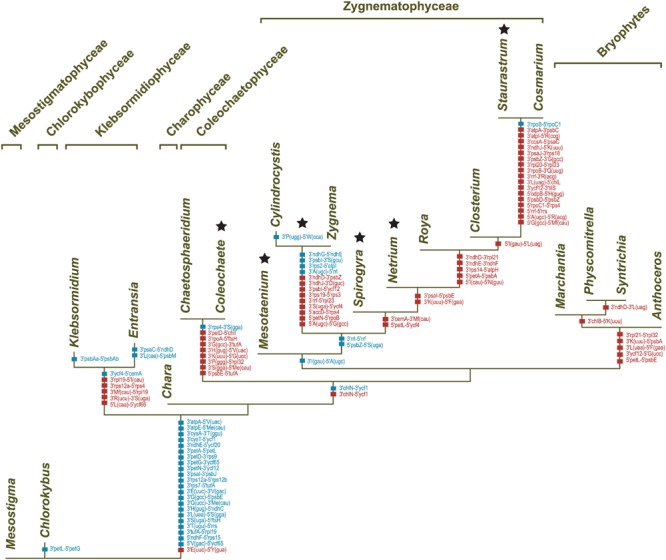
**Synapomorphic gains and losses of gene pairs in streptophyte chloroplast genomes.** These events were inferred in MacClade v4.08 by mapping gene pairs on the streptophyte topology shown in **Figure 6**. The characters denoted by red squares represent synapomorphic gains, whereas those denoted by blue squares represent synapomorphic losses. Note that gene pairs in which at least one gene is missing due to gene loss were not taken into account. Stars denote the six distinct lineages with IR-lacking chloroplast genomes.

Comparative analyses of gene organization were carried out using four complementary approaches. First, syntenic regions were identified in pairwise genome comparisons (**Figures 1–4**). Second, we compared the gene partitioning patterns of the IR-containing genomes and examined whether the genes found in the IR, SSC and LSC are also clustered in IR-lacking genomes (**Figure 10**). Third, phylogenetic trees based on gene order were inferred using the MLGO web server (Hu et al., 2014) and MGR v2.03 (Bourque and Pevzner, 2002): MLGO reconstructs ML phylogenies based on gene adjacency, whereas MGR estimates the number of reversals required to interconvert gene order in pairs of genomes and construct tree topologies based on rearrangement distance (**Figure 11**). The data set analyzed with MLGO contained all standard genes in each genome (including both copies of duplicated genes), while the data set analyzed with MGR was restricted to the 89 genes common to all compared genomes. Finally, the presence/absence of signed gene pairs in three or more genomes (**Figure 12**) were coded as binary Dollo characters and the gene pairs representing synapomorphic gains and losses were mapped on the streptophyte phylogeny reported in this study using MacClade v4.08 (**Figure 13**). The Dollo principle assumes that characters can be lost independently in several evolutionary lineages but cannot be regained. Because inversion endpoints have been shown to be reutilized multiple times in land plant chloroplast genomes experiencing frequent rearrangements (Jansen and Ruhlman, 2012), coding of gene pairs as Dollo characters might not be fully justified. For this reason, we have also coded gene pairs as unordered (Fitch parsimony) or ordered (Wagner parsimony) characters and found no difference in the evolutionary scenarios we inferred.

Although gene order is highly variable among streptophyte algal genomes, the branching order inferred with MLGO is entirely congruent with the phylogeny based on gene and protein sequences (**Figures 6** and **11**). The reversal distances estimated by MGR reveal that the chloroplast genomes of the bryophytes, Coleochaetophyceae and especially *Chara* are the least rearranged relative to those of *Mesostigma* and *Chlorokybus*, and that they are followed closely by the klebsormidiophycean genomes. By comparison, the Zygnematophyceae display very long branches indicative of massive gene shuﬄing. Of the examined zygnematophyceans, the representative of the earliest-diverging lineage (*Mesotaenium*) boasts the least rearranged genome.

The *Coleochaete* and *Chaetosphaeridium* cpDNAs share nine blocks of sequences containing 116 of their 117 and 125 conserved genes, respectively (**Figure 2**). Only eight reversals are required to interconvert the gene order of these two genomes. Following the loss of the IR in the *Coleochaete* lineage, the partitioning of genes between the SSC and LSC regions has been barely affected (**Figure 10**); in other words, the genes corresponding to those located 5′ and 3′ of the rRNA operon in the *Chaetosphaeridium* genome have largely remained clustered in *Coleochaete*. Three of the eight genes missing in *Coleochaete* (*rps14, tilS* and *ccsA*) correspond to breakpoints between syntenic blocks while the remaining, which are all part of ribosomal protein operons, have been cleanly excised from internal regions of syntenic blocks (**Figures 2** and **12**).

The *Klebsormidium flaccidum* and *Entransia* genomes display 26 syntenic blocks containing 85 of their 114 and 118 conserved genes, respectively (**Figure 1**). The vastly expanded IRs of these algae differ considerably from one another and from streptophyte IRs displaying an ancestral organization (i.e., those of *Mesostigma, Chlorokybus, Chara*, coleochaetophyceans and bryophytes) with respect to gene content (**Figure 10**). Of the 32 and 44 genes present in the *Klebsormidium* and *Entransia* IRs, respectively, only 14 are shared besides the five genes making up the rRNA operon; however, all genes typically found 5′ and 3′ of the rRNA operon in ancestral IR-containing genomes, except for *tufA* in *Klebsormidium*, have remained ancestrally segregated. Eight of the 22 genes lost in the lineages leading to *Klebsormidium* and *Entransia* [*accD, minD, psaM, rpl12, rpl32, tufA, trnV*(uac) and *ycf65*] correspond to breakpoints between synteny blocks, while the others have been cleanly excised from internal regions of synteny blocks (**Figures 1** and **12**). Gene rearrangements have disrupted some ancestral gene clusters, e.g., *rpoC2* is no longer adjacent to *rps2* and *atpI* is no longer beside *atpH* (**Figure 12**).

The chloroplast genomes of the Zygnematophyceae exhibit a wide range of divergence at the gene order level (**Figure 11**). The *Cosmarium* and *Staurastrum* genomes are the most similar, with six syntenic blocks containing all 122 encoded genes except *trnS*(uga) (**Figure 4**). All investigated zygnematophycean genomes feature a disrupted rRNA operon; two, three or four breakage sites are observed depending on the species (**Figure 12**). Like the IRs of the *Roya* species, those of *Cosmarium* and *Closterium* harbor two to four tRNA genes in addition to the genes composing the rRNA operon (**Figure 10**). Of these tRNA genes, only *trnR*(acg) is shared with the IRs of streptophyte algal cpDNAs having an ancestral partitioning pattern. All the genes ancestrally located in the SSC and LSC regions, with the single exception of *trnN*(guu), still form ancestrally segregated groups in the *Roya, Cosmarium*, and *Closterium* genomes (**Figure 10**). In contrast, reshuﬄing of gene order in the *Mesotaenium, Zygnema* + *Cylindrocystis* and *Spirogyra* lineages led to extensive dispersal, throughout the genome, of the IR-encoded genes and of the genes typically found in the SSC and LSC regions of ancestral streptophyte IR-containing cpDNAs (**Figure 10**).

Several gene pairs representing synapomorphic signatures of distinct lineages were lost or acquired before the Klebsormidiophyceae, Coleochaetophyceae and bryophyte lineage each arose. But just a single synapomorphy, corresponding to loss of linkage between the *trnI*(gau) and *trnA*(ugc) genes in the rDNA operon, unites the Zygnematophyceae (**Figure 13**). Following the emergence of the *Mesotaenium* lineage, two additional gene rearrangements occurred in the common ancestor of the remaining zygnematophyceans: the rDNA operon was broken at a second site (between *rrl* and *rrf*) and the ancestral pair *5′psbZ*-5*′trnS*(uga) was lost. Reversal of the *ycf1* gene in the ancestral 3*′chlN-*3*′ycf1* pair is a unique gene rearrangement shared by all Coleochaetophyceae, Zygnematophyceae and bryophytes. In contrast, no synapomorphic loss of gene pairs accompanied the emergence of the Charophyceae.

## Discussion

The comparative analyses presented in this study, which include a sampling of nine additional chloroplast genomes from the Klebsormidiophyceae, Coleochaetophyceae and Zygnematophyceae, alter markedly our view of chloroplast genome evolution in streptophytes. Our results clearly indicate that the chloroplast genome is evolving in a dynamic fashion not only in the Zygnematophyceae but also in the Klebsormidiophyceae and Coleochaetophyceae. The diversity of genomic structures and organizations found in these three classes is reminiscent of the diversity that has recently been reported for various classes of the Chlorophyta (Brouard et al., 2010; Lemieux et al., 2014; Leliaert and Lopez-Bautista, 2015; Turmel et al., 2015), and contrasts with the extremely conservative evolutionary trend observed in most land plants (Wicke et al., 2011; Jansen and Ruhlman, 2012; Ruhlman and Jansen, 2014). In the following sections, we highlight the evolutionary trends observed in each streptophyte algal class.

The phylogenies we inferred from 88 chloroplast protein-coding genes and proteins are congruent with recent phylogenomic studies indicating that the Zygnematophyceae is sister to land plants (Wodniok et al., 2011; Laurin-Lemay et al., 2012; Timme et al., 2012; Zhong et al., 2013; Wickett et al., 2014). The relationships among the members of the Zygnematophyceae are consistent with the phylogeny reported by Gontcharov et al. (2004). Moreover, the differences in branching order of the bryophyte lineages between the protein and gene trees are in agreement with previously reported phylogenomic studies (Cox et al., 2014; Wickett et al., 2014).

### Mesostigmatophyceae and Chlorokybophyceae

The *Mesostigma* and *Chlorokybus* chloroplast genomes are the most rich in ancestral traits among the Viridiplantae (Lemieux et al., 2000, 2007). They feature the most extensive gene content, are almost devoid of introns, contain many operons typically found in cyanobacteria, and have retained a quadripartite architecture with a pattern of gene partitioning that closely resembles those found in early diverging members of the Chlorophyta (e.g., *Nephroselmis*; Turmel et al., 1999b; Lemieux et al., 2014 and *Pyramimonas*; Turmel et al., 2009) (**Table 1**; **Figure 10**). These ancestral features mirror the deep-branching positions of *Mesostigma* and *Chlorokybus* and illustrate the great structural stability of the chloroplast genome in the clade uniting these algae.

### Klebsormidiophyceae

The *Entransia* and *Klebsormidium flaccidum* chloroplast genomes have retained a quadripartite architecture, but their IRs are greatly enlarged and include many genes typically found in the SSC and LSC regions (**Figures 1, 5**, and **10**). Reconstruction of the ancestral genome reveals that the IR underwent considerable expansion toward the SSC region before the split of the two klebsormidiophycean lineages and that it expanded predominantly toward the LSC region following this divergence. At 61 kb, the *Entransia* IR is the largest known among the green algae examined so far. Compared to the *Klebsormidium flaccidum* IR, it contains twice as many genes of LSC origin but fewer genes of SSC origin, suggesting that shifts of both the IR/SSC and IR/LSC junctions are on-going events in the Klebsormidiophyceae. Sampling of additional taxa from this class should provide more information on the directionality and extent of these shifts in various lineages. Like klebsormidiophycean cpDNAs, the chloroplast genomes of the chlorodendrophycean green algae *Scherffelia* and *Tetraselmis* feature enlarged IRs with a rich gene content, but the ancestral partitioning pattern has not been maintained (Turmel et al., 2016).

Thirty standard chloroplast genes were lost during the evolution of the Klebsormidiophyceae; losses of *ndhK, rpl14, rpl16, rps3, trnG*(ucc), *trnT*(ggu), *trnT*(ugu), *trnV*(gac) and *trnV*(uac), in particular, are unique among streptophyte algae (**Figure 7**). As a consequence of the substantial losses of *trn* gene, the complement of tRNAs encoded in klebsormidiophycean chloroplast genomes is not sufficient to decode the entire set of codons found in these genomes. For example, there is no chloroplast-encoded tRNA^Thr^ in *Klebsormidium* and *Entransia*, and no chloroplast-encoded tRNA^V al^ in *Klebsormidium*. It thus appears that these missing tRNAs are imported from the cytosol into the chloroplast. Import of nuclear-encoded tRNAs into plastids has previously been suggested for non-photosynthetic land plants (Wicke et al., 2011).

Klebsormidiophycean chloroplast genomes contain an abundance of introns. Civan et al. (2014) previously inferred that the common ancestor of the Klebsormidiophyceae and its sister lineages (branch I in **Figure 9**) shared five group II introns with extant land plants. To this set of early acquired introns, we must now add the *rpl16*_9 and *trnV*(uac)_37 introns, which we identified in the *Entransia* genome. Although the *Klebsormidium flaccidum* and *Entransia* chloroplast genomes contain a large number of lineage-specific group II introns (seven are unique to *Klebsormidium* and four are unique to *Entransia*), they have no intron insertion sites in common other than those shared with land plants, thus suggesting that the lineage-specific introns were mostly acquired through intragenomic proliferation of founding introns.

It is intriguing that the *tufA* gene resides in the SSC region rather than in the LSC region in the *Klebsormidium* chloroplast genome and that it is entirely missing in the *Entransia* genome. In *Mesostigma, Chlorokybus* and most chlorophyte cpDNAs, *tufA* is part of the *str* operon, which also comprises *rps12* and *rps7* (transcription order is 5*′*-*rps12-rps7-tufA*-3*′*). It appears that breakage of this operon through the acquisition of a *trans*-spliced group II intron at site 114 of *rps12* soon after the divergence of the *Mesostigma* + *Chlorokybus* clade led to relocation of *rps12* exon 1 outside the operon and ultimately to loss of linkage between *rps7* and *tufA*, which resulted in transfer of *tufA* to the SSC region in *Klebsormidium* and to the complete loss of this gene in *Entransia*. There is also no linkage between *rps7* and *tufA* in the *Chara* and *Chaetosphaeridium* genomes, and both *rps12* and *rps7* are completely missing in *Coleochaete*.

### Charophyceae

Only the chloroplast genome sequence of *Chara vulgaris* is currently available for the Charophyceae (Turmel et al., 2006). Remarkably, this alga has retained the largest degree of ancestral traits among the streptophytes that diverged after the *Mesostigma* + *Chlorokybus* clade. This highly conservative evolutionary trend is apparent at all levels, including overall architecture, gene content, gene partitioning, and gene organization (**Figure 10**). The *Chara* genome is clearly the least rearranged relative to the *Mesostigma* and *Chlorokybus* cpDNAs, as revealed by the short branch length separating these taxa in the MGR tree shown in **Figure 11**. It will be interesting to see if sampling of additional taxa from the Charophyceae will support the notion that the chloroplast genome is evolving at a very slow rate in this class. In this context, it is noteworthy that the mitochondrial genomes of the distantly related charophycean algae *Chara vulgaris* and *Nitella hyalina* contain the same gene complement and display exactly the same gene order (Turmel et al., 2013).

### Coleochaetophyceae

Our chloroplast genome analyses of a second representative of the Coleochaetophyceae unveiled a less conservative evolutionary history than previously thought for this class. Genome streamlining appears to be the main evolutionary force in the lineage leading to *Coleochaete*. The *Coleochaete* genome is both the smallest and most compact among the streptophyte algal cpDNAs investigated so far (**Table 1**). Unlike the *Chaetosphaeridium* cpDNA (Turmel et al., 2002), it lacks an IR and several standard genes. Notably, losses of four ribosomal protein-coding genes (*rps4, rps7, rps12*, and *rps14*) represent unique events in the evolutionary scenario we inferred for streptophyte algae (**Figure 7**). Otherwise, gene organization has been highly preserved in *Coleochaete* and *Chaetosphaeridium* and the genes usually present in the IR, SSC and LSC regions have retained an ancestral partitioning pattern in the *Coleochaete* genome (**Figures 10** and **11**).

The *tufA* gene is evolving at a fast pace in the Coleochaetophyceae (**Figure 8**). Considering that this gene has completely disappeared from the chloroplast genome and is most probably nuclear-encoded in all streptophyte lineages that evolved after the divergence of the Coleochaetophyceae (**Figure 7**), it is possible that the chloroplast *tufA* sequences identified in the Coleochaetophyceae do not encode the functional elongation factor EF-Tu; instead, a nuclear gene product might play this role in protein synthesis. This hypothesis was proposed earlier by Baldauf et al. (1990) who reported that the chloroplast *tufA* sequence of *Coleochaete orbicularis* is unusually divergent and differs considerably at what was otherwise conserved amino acid positions. These authors speculated that, despite the presence of numerous mutations, long-term maintenance of an intact ORF at the *Coleochaete tufA* locus might be the result of selection to retain less constrained subsets of the original EF-Tu functions. This hypothesis is attractive, considering that EF-Tu has been shown to play an important role in cell shape maintenance in *Bacillus subtilis* through direct interaction with MreB (Defeu Soufo et al., 2010), a protein involved in septum synthesis and cell division (Fenton and Gerdes, 2013); however, it is not supported by our finding that there was no positive selection across the *tufA* sequence in the Coleochaetophyceae. Putative nuclear copies of *tufA* were detected in *Coleochaete orbicularis* by Southern blot analysis (Baldauf et al., 1990), and our BLASP searches against the 1000 Plants (oneKP) database^8^ using the *Chara* chloroplast *tufA* sequence as query identified highly similar sequences (*E*-value threshold of 0.0) that contain all functional domains of EF-Tu in the RNA-seq assemblies of *Coleochaete scutata* (VQBJ-2010477), *Coleochaete irregularis* (QPDY-2029449) and *Chaetosphaeridium globosum* (DRGY-2007378). Taken together, these observations support the notion that the nucleus houses the functional coding sequence for the chloroplast EF-Tu and that the divergent *tufA* sequence in the chloroplast genome is undergoing pseudogenization.

### Zygnematophyceae

Previous studies revealed that the zygnematophycean chloroplast genome is highly variable in overall structure, gene order and intron content (Turmel et al., 2005, 2007; Civan et al., 2014). The comparative genome analyses reported here, which include six additional taxa sampled from the Zygnematales and Desmidiales, underscore the exceptionally dynamic evolution of this genome.

To account for the presence of an IR in one of the four zygnematophycean taxa they examined (*Roya*), Civan et al. (2014) proposed that the IR was either lost three times or gained once *de novo* during the diversification of the Zygnematophyceae. Our finding that the cpDNAs of the desmidialeans *Closterium* and *Cosmarium* also have an IR suggests that the IR was lost a minimum of five times (**Figures 5** and **13**). It is unlikely that the IR was acquired *de novo* on one or more independent occasions because the ancestral gene partitioning pattern has been retained in late-diverging zygnematophyceans with IR-less chloroplast genomes (*Staurastrum* and *Netrium*). Indeed, considering that the genes originally present in the IR and SSC regions were dispersed throughout the genome as a result of high frequency gene rearrangements in early diverging lineages of the Zygnematophyceae (**Figure 10**), it is difficult to envision that the region containing all the genes encoded by the rDNA operon became duplicated at exactly the same site as the ancestral LSC/SSC junction in the common ancestor of *Roya, Closterium, Staurastrum*, and *Cosmarium* and that the ancestral gene partitions were restored concomitantly (**Figure 6**). The notion that the quadripartite structure was eliminated multiple times in the Zygnematophyceae predicts that future studies with a broader taxon sampling of this class will uncover early diverging taxa harboring IR-containing chloroplast genomes. Independent losses of the IR also took place in other lineages of the Viridiplantae, including land plants (Wicke et al., 2011; Jansen and Ruhlman, 2012; Ruhlman and Jansen, 2014). The quadripartite structure was eliminated at least four times in prasinophytes (Turmel et al., 2009; Lemieux et al., 2014), seven times in the Trebouxiophyceae (de Cambiaire et al., 2007; Turmel et al., 2015), twice in the Ulvophyceae (Lu et al., 2011; Leliaert and Lopez-Bautista, 2015; Melton et al., 2015) and once in the Chlorophyceae (Bélanger et al., 2006; Brouard et al., 2010, 2011). In the case of the Chlorophyceae, the reported loss unites two major lineages of the OCC clade: the Chaetophorales and Chaetopeltidales (Brouard et al., 2010).

The long branches leading to the zygnematophycean taxa in the MGR tree (**Figure 11**) indicate that the chloroplast genome underwent more extensive gene scrambling in the Zygnematophyceae than in any other streptophyte algal classes. These gene rearrangements were accompanied by the disruption of several ancestral clusters, including the rDNA operon (**Figure 13**). The latter operon was broken at four distinct sites, three of which are associated with synapomorphic losses of gene pairs: (1) the *trnI*(gau)-*trnA*(ugc) pair in the common ancestor of all zygnematophyceans, (2) the *rrl*-*rrf* pair following the divergence of *Mesotaenium* and (3) the *rrl-rrf* pair in the common ancestor of *Zygnema* and *Cylindrocystis*. The fourth site, located between *rrs* and *trnI*(gau), was disrupted along the branches leading to *Mesotaenium*, to the common ancestor of *Zygnema* and *Cylindrocystis*, and to the common ancestor of *Netrium* and its sister clade. Disruptions of the chloroplast rDNA operon are rare events among viridiplants but have been reported in some chlorophyte lineages featuring highly rearranged genomes (de Cambiaire et al., 2007; Turmel et al., 2009, 2015; Lu et al., 2011; Lemieux et al., 2014; Leliaert and Lopez-Bautista, 2015).

In land plants, IR loss and/or acquisition of short dispersed repeats have been associated with an increased rate of genome rearrangements (Palmer, 1991; Wicke et al., 2011; Jansen and Ruhlman, 2012; Weng et al., 2014), but these factors are unlikely to be the main force driving genome rearrangements in zygnematophyceans. Although gene order in the *Zygnema* and *Cylindrocystis* IR-less genomes was reconfigured at a faster rate compared to their IR-containing homologs, this is not the case for other zygnematophycean lineages (*Mesotaenium, Spirogyra*, and *Netrium*) that also feature IR-less genomes but much shorter branches (**Figure 11**). Similarly, the extent of gene rearrangements and repeat contents are weakly correlated; zygnematophycean genomes are generally poor in short dispersed repeats and those having the highest proportion of these elements (*Cosmarium* and *Closterium* cpDNAs) show minor differences in gene rearrangements (**Figure 11**) compared to their closest relatives having much fewer repeats (*Stauratrum* and *Roya*).

In agreement with the study of Civan et al. (2014), we inferred that the common ancestor of zygnemataleans harbored all the 21 group II introns usually present in land plant genomes, except those in *trnI*(gau), a gene that was part of the ancestral rDNA operon, and in *trnV*(uac). Just three introns — *rpl16*_9, *rps12*_114 and *trnG*(ucc)_23 — were retained in all ten examined taxa. While Civan et al. (2014) predicted 20 independent events of intron loss in the Zygnematophyceae, we scored 43 losses in our study (**Figure 9**). Two introns were lost only once (those at sites 18 and 31 in **Figure 9**), six were lost twice (sites 2, 5, 7, 9, 26, 29), and the remaining introns on three, four or five occasions. The underlying cause of this intron instability remains unclear. It has been previously speculated that intron losses could be the result of retroposition events (reverse transcription of a spliced RNA copy, followed by recombination-dependent insertion into the genome) and that the protein encoded by the *trans*-spliced *rps12*_114 intron in several zygnematophycean lineages could provide the RT activity required for these events (Turmel et al., 2005; Civan et al., 2014). Although RT-mediated intron loss is a mechanism that is very efficient in removing introns (Cohen et al., 2012), very few zygnematophycean genomes actually encode a protein with this activity. Our BLAST analyses of group II intron-encoded ORFs revealed that only the *orf643* in the *psbE*_71 group II intron of *Netrium* (an intron unique to this alga) codes for a putative RT; all other intron-encoded ORFs were found to contain an intron maturase domain, including the *Staurastrum orf404* that we incorrectly annotated as a RT gene in a previous report (Turmel et al., 2005). These observations, however, do not necessarily invalidate the retroposition hypothesis for intron removal in the Zygnematophyceae, as RT genes might have been present in ancestral chloroplast genomes. Alternatively, RT activities of mitochondrial or nuclear origin might also be invoked to support this hypothesis. The finding of a group II intron-encoded RT in the mitochondrial *cox2* gene of *Closterium baillyanum* is consistent with this idea (Turmel et al., 2013).

Putative gains of foreign genes by the chloroplast genome have been documented for several green algal lineages: prasinophytes (Turmel et al., 2009; Lemieux et al., 2014), Trebouxiophyceae (Turmel et al., 2015), Ulvophyceae (Leliaert and Lopez-Bautista, 2015), Chlorophyceae (Brouard et al., 2008) and streptophyte algae (Civan et al., 2014). Here we report further evidence that genes encoding integrases/recombinases (Int) and DNA primases of phage/viral origin were transferred to the chloroplast during the evolution of the Zygnematophyceae (**Table 2**). Putative Int-encoding genes have been also identified in the mitochondrial genomes of various streptophytes: *Klebsormidium flaccidum* (*orf374* in GenBank:KP165386), *Chaetosphaeridium globosum* (Turmel et al., 2002*), Roya obtusa* (Turmel et al., 2013) and the lycophyte *Phlegmariurus squarrosus* (*orf233* in GenBank JQ002659). Inter-organellar gene transfers might account for the presence of these genes in both the chloroplast and mitochondria.

Early insertions of viral genes in the IR of the zygnematophycean chloroplast genome might have contributed to the instability of the IR. Civan et al. (2014) proposed that both cpDNA-encoded RT and Int activities have shaped this genome. Given that these activities are essential components of the replicative machinery of retroelements, they speculated that invasion of retroviruses and/or retrotransposons in the chloroplasts of early diverging zygnematophyceans triggered massive genome rearrangements. However, this scenario is not consistent with the findings that the chloroplast-encoded Int sequences uncovered in the zygnematophyceans investigated so far more likely originate from phages/viruses than retroelements and that only *Netrium* carries a chloroplast-encoded RT. As proposed for other streptophyte lineages with unusually rearranged chloroplast genomes (Weng et al., 2014; Zhang et al., 2016), it appears more likely that nuclear-encoded, plastid-targeted genes involved in DNA replication, recombination, and repair (and also perhaps in reverse transcription) played a major important role in reshuﬄing the zygnematophycean chloroplast genome.

## Author Contributions

CL and MT conceived and designed the research. CL and CO performed the research. CL, CO, and MT analyzed the data. MT and CL wrote the paper.

## Conflict of Interest Statement

The authors declare that the research was conducted in the absence of any commercial or financial relationships that could be construed as a potential conflict of interest.

## Footnotes

1 http://www.uni-goettingen.de/de/184982.html

2 http://utex.org/

3 http://gqinnovationcenter.com/index.aspx

4 http://pag.ibis.ulaval.ca/seq/en/

5 http://pag.ibis.ulaval.ca/seq/en/

6 http://www.repeatmasker.org/

7 http://www.phrap.org/

8 https://www.bioinfodata.org/Blast4OneKP/home
