# Supplementary material for: Comparative Chloroplast Genome Analyses of Streptophyte Green Algae Uncover Major Structural Alterations in the Klebsormidiophyceae, Coleochaetophyceae and Zygnematophyceae
Source: Front Plant Sci. 2016 May 24;7:697. doi: 10.3389/fpls.2016.00697 (PMC4877394; doi:10.3389/fpls.2016.00697)
Supplement: Supplementary file 1 [file Image_1.PDF]

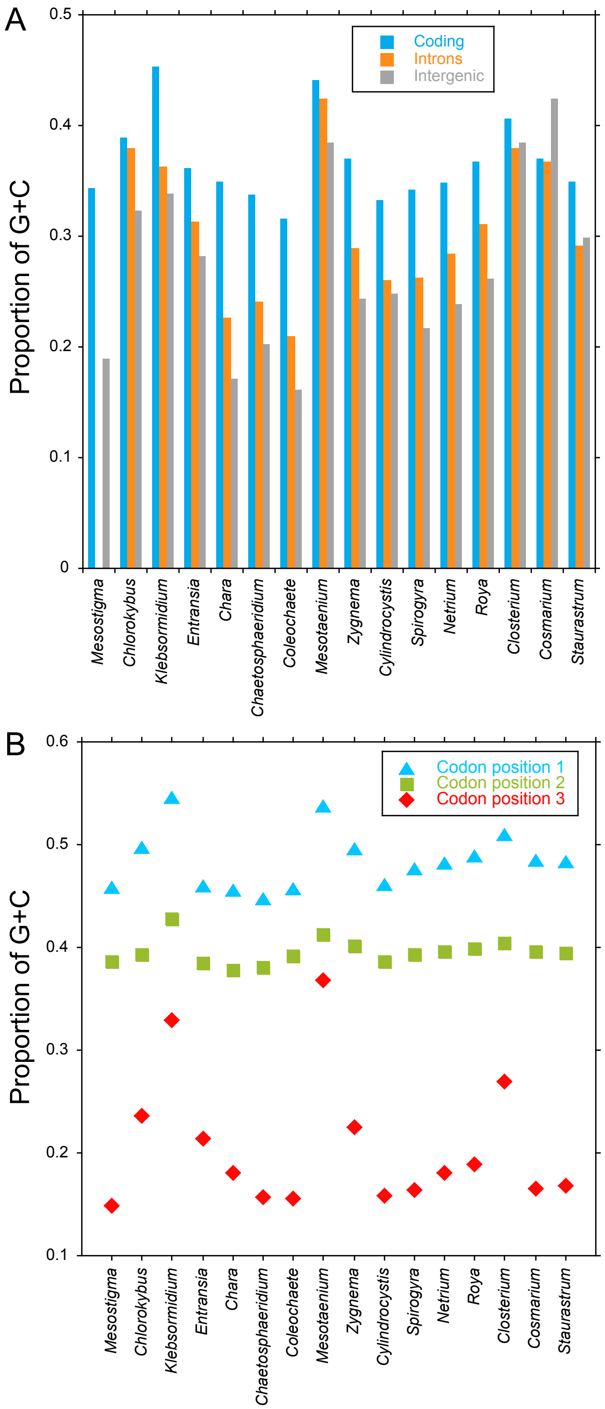

Supplementary FIGURE 1 | G+C content of: (A) coding regions, introns and intergenic spacers among 16 streptophyte chloroplast genomes, and (B) first, second and third codon positions of protein-coding genes. G+C values at each codon position were calculated using the PCG123 nucleotide data set (88 genes, 19,177 codons).
