# Supplementary material for: Comparative Chloroplast Genome Analyses of Streptophyte Green Algae Uncover Major Structural Alterations in the Klebsormidiophyceae, Coleochaetophyceae and Zygnematophyceae
Source: Front Plant Sci. 2016 May 24;7:697. doi: 10.3389/fpls.2016.00697 (PMC4877394; doi:10.3389/fpls.2016.00697)
Supplement: Supplementary file 2 [file Image_2.PDF]

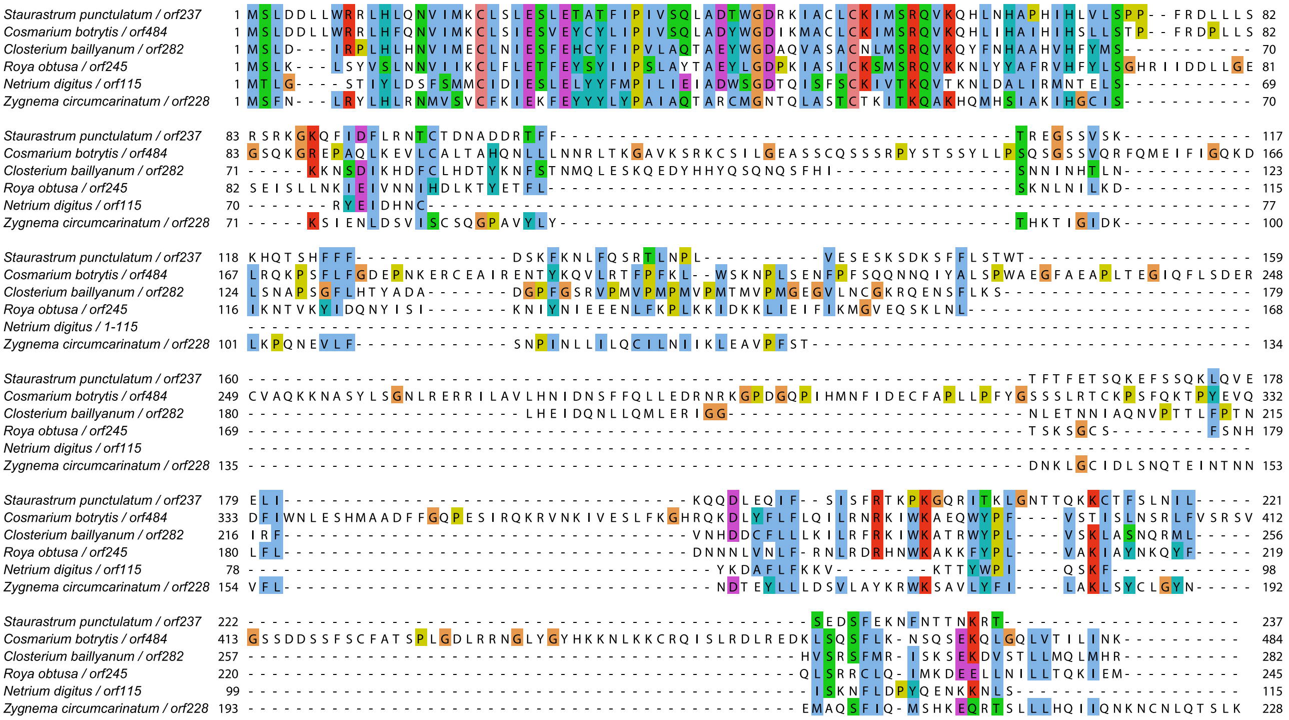

Supplementary FIGURE 2 | Alignment of translated ORFs located between *psbJ* and *petA* in zygnematalean chloroplast genomes. The genomic coordinates of the ORF sequences corresponding to these proteins are as follows: *Staurastrum punctulatum orf237* (63662..62949 in GenBank:NC_008116); *Cosmarium botrytis orf484* (33939..32491 in GenBank:KU646492); *Closterium baillyanum orf282* (95263..94421 in GenBank:KU646494); *Roya obtusa orf245* (23487..22750 in GenBank:KU646496); *Netrium digitus orf115* (92784..93131 in GenBank:KU646491); *Zygnema circumcarinatum orf228* (96009..96695 in GenBank:NC_008117).
